# Influence of Macro-Topography on Damage Tolerance and Fracture Toughness of 0.1 wt % Multi-Layer Graphene/Clay-Epoxy Nanocomposites

**DOI:** 10.3390/polym8070239

**Published:** 2016-07-01

**Authors:** Rasheed Atif, Fawad Inam

**Affiliations:** Faculty of Engineering and Environment, Department of Mechanical and Construction Engineering, Northumbria University, Newcastle upon Tyne NE1 8ST, UK; aatif.rasheed@northumbria.ac.uk

**Keywords:** topography, mechanical properties, fracture toughness, 0.1 wt % MLG/clay-epoxy nanocomposites, dynamic mechanical properties

## Abstract

Influence of topographical features on mechanical properties of 0.1 wt % Multi-Layer Graphene (MLG)/clay-epoxy nanocomposites has been studied. Three different compositions were made: (1) 0.1 wt % MLG-EP; (2) 0.1 wt % clay-EP and (3) 0.05 wt % MLG-0.05 wt % clay-EP. The objective of making hybrid nanocomposites was to determine whether synergistic effects are prominent at low weight fraction of 0.1 wt % causing an improvement in mechanical properties. The topographical features studied include waviness (*W*_a_), roughness average (*R*_a_), root mean square value (*R*_q_) and maximum roughness height (*R*_max_ or *R*_z_). The *R*_z_ of as-cast 0.1 wt % MLG-EP, clay-EP and 0.05 wt % MLG-0.05 wt % clay-EP nanocomposites were 43.52, 48.43 and 41.8 µm respectively. A decrease in *R*_z_ values was observed by treating the samples with velvet cloth and abrasive paper 1200P while increased by treating with abrasive papers 320P and 60P. A weight loss of up to 16% was observed in samples after the treatment with the abrasive papers. It was observed that MLG is more effective in improving the mechanical properties of epoxy than nanoclay. In addition, no significant improvement in mechanical properties was observed in hybrid nanocomposites indicating that 0.1 wt % is not sufficient to generate conspicuous synergistic effects.

## 1. Introduction

Due to the tribological protection offered by stiff technical polymers such as epoxy, there is increased interest to employ them in mechanical engineering applications [1,2,3,4]. To grasp a phenomenological understanding of tribology and fracture mechanics, it is of foremost importance to study the interplay between topographical features and bulk properties [5]. To improve the wear resistance of monolithic polymers, surface coatings are applied. It is because the preferential growth of crystallites in subsequent deposition closes the cracks and gives the option to tailor the topographical features as per the design/service requirements [6,7,8,9]. Various coating techniques include galvanic/electrochemical deposition and plasma and thermal spraying that can yield thick coatings of high load support [10].

Although efficient adhesion strength between coating and substrate may be achieved in as-coated samples, delamination takes place when subjected to external loading. It is because the coatings have too high stiffness and too low plastic deformability to follow the substrate deformation. This disparity may be exacerbated in the presence of thermal stresses or elevated temperatures due to a disparate coefficient of thermal expansion (CTE) of coating and the substrate. For example, epoxy may show 10 times more thermal expansion than most of the thin film materials studied [10]. Alternatively, polymer coatings on polymer substrate may have comparable stiffness and CTE; however they fail in tribological applications. Therefore, even if coated it is highly likely that the polymers will suffer from wear in applications where sliding contact is inevitable. Hence, it becomes essential to improve the properties of monolithic polymers for tribological applications. The tribological properties of polymers can be enhanced with the incorporation of (nano-) reinforcement such as metallic oxides [11,12,13], clays [14,15,16], carbon nanotubes (CNTs) [17,18,19] and other carbonaceous materials [20,21,22].

The topography is not only important in monolithic polymers, but also in case of Polymer Matrix Composites (PMCs) [23,24,25,26]. Epoxy is an excellent matrix for composites because of its many features superior to that of the competition, including but not limited to handling characteristics, improvement in composite mechanical properties, acceptable cost and processing flexibility [27]. A plethora of research has been dedicated to improve the performance of polymer nanocomposites. Gao *et al.* have shown that interfacial interactions and mechanical properties of carbon fiber-epoxy composites can be improved by increasing the surface roughness of the filler [28]. They also showed that surface roughness in a few tens of nanometer scale does not contribute significantly in increasing the interfacial adhesion from the “mechanical interlocking” [28].

Surfaces can be made rough or porous to enhance the extent of mechanical interlocking [29]. Karger-Kocsis *et al.* have reported that hierarchical and hairy fillers have high surface area and capillary wetting by the polymers [30]. The textured fillers also exhibit mechanical interlocking with the polymers and cause local reinforcement of the fiber-matrix interphase [30]. Moon and Jang studied the mechanical interlocking and wetting at the interface between argon plasma treated ultra-high modulus polyethylene (UHMPE) fiber reinforced vinylester resin composite [31]. They observed a significant increase in interlaminar shear strength. It has been shown that plasma etching of UHMPE produces micro-pittings on fiber surface and this spongy surface structure helps improve mechanical interlocking with the polymer matrix and causes a significant increase in interlaminar shear strength [32,33,34,35]. The topography can be controlled during synthesis such as using a combination of UV lithography and electro-deposition [36]. The topographical features may also be tailored by texturing the mold surface when the production route is the casting.

The tribology primarily deals with the surface condition and topography. The various features of topography include: (1) surface roughness; (2) surface waviness; (3) surface form and (4) lay [29]. As various factors influence topographical features, therefore it is conventional to study them separately during the analysis. In general, component performance related to topography (e.g., friction reflectivity, wear characteristics, lubrication properties and resistance to stress failure) is studied evaluating the surface roughness parameter. And component performance related to abrasive tool (e.g., vibration or noise generation) is studied by analyzing the surface waviness parameter [29].

Any regular or irregular spacing on the surface tend to form a texture or pattern [29]. The surface textures are formed during the casting, manufacturing or machining processes. Another important factor is microstructure of the material as internal stresses released after machining, can also contribute to surface deformation and may form a specific topography. The machining processes can significantly influence the topography. A major factor is the action of the abrasive tool on the material. Elements such as tool speed, feed, shape and cutting fluids can affect the topography. Other influential factors can be instability of the abrasive tool due to chatter or imbalance in the grinding wheel and errors in the machine tool guideway [29].

The primary reason to study the topographical features is to try and forecast the performance of the system. For example, the surface of a bearing should be textured such that it allows lubricant to be retained in small pockets and at the same time allows the bearing movement with a minimum of friction. If the surface has high roughness, wear will be expedited; however if the surface has low roughness, poor lubrication and seizure may take place. Therefore, a compromise between smoothness and roughness is essential to maintain. The other reason to measure the topographical features is to control the manufacturing process as the operator can detect variation in surface finish and adjust the controllables to ensure that the process remains in limit [29].

A simple and common method to measure topographical features is the surface texture recorder [29]. The stylus is moved across the surface with the help of a guiding mechanism to produce the “traced profile”, which is produced by the interaction of the stylus with the surface of the sample. The transducer generates a signal which is produced from the difference between a “reference profile” or “datum profile” and the traced profile. The transducer signal is converted into a digital signal using an analog-to-digital converter. At this point, the transducer contains only the vertical or Z-component of the profile. The traversing component generates the horizontal or X-component which is combined with the Z-component to acquire the “total profile”. The total profile is then filtered to omit unnecessary information which generates a “primary profile”. The filtering techniques can further be employed to separate the waviness, roughness and form features of the surface [29].

For effective analysis of topographical features, the obtained profile needs to be analyzed according to internationally recognized mathematical formulae which are called parameters. A certain number can be given to a certain aspect of topographical features to compare it with another pattern or reference and to remove the need for subjective operator assessment. A single parameter is not enough to characterize the topography completely. Therefore, multiple parameters are usually used. The parameters can be divided into four basic types: (1) Amplitude parameters measure the vertical characteristics of topography; (2) Spacing parameters measure the irregularity spacings along the surface, regardless of the amplitude of these irregularities; (3) Hybrid parameters measure a combination of the spacing and amplitude of the surface irregularities; (4) Extended parameters are not only defined by the profile data and require further attributes or inputs [29]. The most common use of engineering surfaces is to provide a bearing surface for another component moving relative to it, resulting in wear.

The damage tolerance is the ability of a critical structure to withstand a level of service or manufacturing-induced damage or flaws while maintaining its function [27]. The damage tolerance of aircraft components is necessarily studied to avoid any catastrophic in-flight failure. The damage tolerance tests ensure that the component under inspection does not undergo functional impairment during its service life or within the duration between two scheduled maintenances. The functional impairment is defined as the presence of damage in a part that requires maintenance action [27]. The PMCs have found extensive applications in aerospace, automotive and construction owing to ease of processing and high strength to weight ratio which is an important property required for aerospace applications [37]. Among different polymers, epoxy is the most commonly used thermosetting polymer matrix in PMCs [27].

Addition of hybrid nano-fillers not only improves dispersion states of MLG and CNT in the polymer matrix but synergistic effects also become active that help improve the physical properties of hybrid nanocomposites [38]. Sumfleth *et al.* doped titania into MWNT-epoxy system [38]. They found enhanced CNT dispersion and synergistic effects in these multiphase nanocomposites. Ma *et al.* doped nanoclay into CNT- acrylonitrile butadiene styrene (ABS) system and found enhanced CNT dispersion [38]. Nanoclay also improved CNT dispersion state in CNT-polyamide nanocomposites [38]. Titania can improve mechanical properties of polymers. So, titania is a better option to improve CNT dispersion than block copolymers. Also, large CNT loading can be uniformly dispersed using titania. The addition of nanoparticles in nanocomposites can improve their thermal stability (*T*_g_) which is an important requirement for structural applications [38].

In the current study, topography was modified by treating MLG/clay-EP nanocomposites with abrasive papers of different surface roughness values. Three different compositions were made: (1) 0.1 wt % MLG-EP; (2) 0.1 wt % clay-EP and (3) 0.05 wt % MLG-0.05 wt % clay-EP. The objective of making hybrid nanocomposites was to determine whether synergistic effects are prominent at low weight fraction of 0.1 wt %. An Alicona optical microscope was used to study the topographical features of produced samples. The topographical features, mechanical properties and dynamic mechanical properties of produced nanocomposites were studied. The results showed that topographical features can significantly influence the above-stated properties of 0.1 wt % MLG/clay-EP nanocomposites. In addition, no significant improvement in mechanical properties was observed in hybrid nanocomposites indicating that 0.1 wt % is not sufficient to generate conspicuous synergistic effects.

## 2. Materials and Methods

MLG of 12 nm average thickness and 4.5 µm average lateral size with specific surface area of 80 m^2^/g and purity 99.2% was purchased from Graphene Supermarket (Graphene Supermarket, Calverton, NY, USA). Halloysite nanoclay was used as second filler and purchased from Sigma-Aldrich (Sigma-Aldrich, Dorset, UK). The diameter is between 30–70 nm with length 1–4 µm and has a tube-like morphology. The density of halloysite nanoclay is 2.53 g/cm^3^ and surface area is 64 m^2^/g. It has low electrical and thermal conductivities and strong hydrogen interactions, on account of which the inner hydroxyl groups show greater stability than the surface hydroxyl groups in halloysite. The tube-like morphology, high aspect ratio and low percolation make halloysite nanoclay a potential reinforcement for epoxy and other polymers. Bisphenol A-epichlorohydrin based epoxy having a density of ~1.3 g/cm^3^ and dimethylbenzylamine isophorone diamine based low viscosity fast curing hardener with ~1.1 g/cm^3^ density were used in the current study and purchased from Polyfibre, Birmingham, UK. This epoxy system is a multi-purpose resin offering good all-round properties with the epoxy group content of 4.76–5.25 mol/kg. The viscosity of liquid epoxy and hardener are 12,000–15,000 cps and 45 cps at room temperature, respectively. To prepare 0.1 wt % MLG-EP samples, the mix proportions are 50 parts by weight of hardener to 100 parts by weight of liquid epoxy. The gelation time of the resin was 43 min at room temperature.

The fillers were dispersed in resin using tip sonication for 3 h. The sonication was carried out using a tip sonicator of 750 W and a frequency of 250 kHz (Vibra-cell model VC 750, Newtown, CT, USA). The operation mode was 70% power with 10 s vibration and 5 s break. Although the sonication was carried out at room temperature, the temperature of the system rose due to the high energy vibration produced by the tip sonicator. The epoxy and hardener were degassed separately for 1 h. The two parts were mixed in epoxy: hardener ratio of 2:1. Following thorough hand mixing for 10 min, vacuum degassing was again carried out for 15 min. The resin was poured into silicone molds (without any release agent) and cured at room temperature for 6 h followed by post-curing at 150 °C overnight to ensure completion of the crosslinking. The top and bottom surfaces of each sample were treated with abrasive papers for 1 min on rotating wheels at rotational speed of 150 rpm.

The densification of samples was calculated according to ASTM Standard D792. The densities of epoxy, hardener and water were, 1.3, 1.1 and 0.9975 g/cm^3^, respectively. Experimental density and densification were calculated using Equations (1) and (2) respectively.

(1) $$\text{Experimental density}=\frac{\text{Weight in Air}}{\text{Weight in Air}\;–\text{Weight in Water}}\times \text{Density of water}$$

(2) $$\text{Densification}\;\left(\%\right)\;=\frac{\text{Experimental Density}}{\text{Theoretical Density}}\;\times 100$$

**Figure 1:**
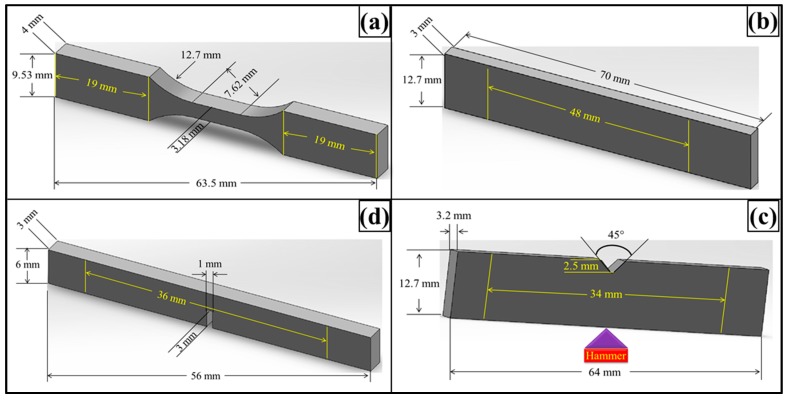
Schematics of mechanical test specimens: (**a**) Tensile; (**b**) Three point bend; (**c**) Charpy impact toughness; (**d**) Fracture toughness test specimens.

The Vickers microhardness test was conducted using Buehler Micromet II (Spectrographic, Bradford, UK) to determine the hardness values of the samples. The load applied was 200 g for 10 s. Tensile, three-point bending and fracture toughness tests were conducted using Instron Universal Testing Machine (Model 3382, Instron, Buckinghamshire, UK). The displacement rate was kept 0.5 mm/min for tensile and fracture toughness tests and 1 mm/min for the three-point bending test. Five specimens were tested for each composition. The schematics of the specimens are shown in Figure 1.

Tensile properties were measured according to ASTM D638 Type-V geometry (ASTM, NY, USA) with a specimen thickness 4 mm. Three-point bending test was conducted according to ASTM D790 with specimen dimensions 3 mm × 12.7 mm× 48 mm. A single-edge-notch three-point bending (SEN-TPB) specimen was used to determine mode-I fracture toughness (*K*_1C_) according to ASTM D5045. The specimen dimensions were 3 mm × 6 mm× 36 mm with a crack of length 3 mm. The notch was made at the mid of sample and tapped to sharpen by a fresh razor blade. The K_1C_ was calculated using Equation (3),

(3) K1C=Pmaxf(aw)BW12

where, *P*_max_ is maximum load of load-displacement curve (N), f(a/w) is constant related to geometry of the sample and was calculated using Equation (4), B is sample thickness (mm), W is sample width (mm) and a is crack length (kept between 0.45 W and 0.55 W). The critical strain energy release rate (G_1C_) was calculated using Equation (5) where *E* is the Young’s modulus obtained from the tensile tests (MPa), and ν is the Poisson’s ratio of the polymer, taken to be 0.35.

(4) f(aw)=[(2+aw){0.0866+4.64(aw)−13.32(aw)2+14.72(aw)3−5.6(aw)4}](1−aw)3/2

(5) $$G_{1c}=\frac{K_{1c}^{2}\left(1-\nu^{2}\right)}{E}$$

Charpy impact toughness test was carried out according to ASTM D6110 (ASTM, NY, USA) using notched specimen with dimensions 3.2 mm× 12.7 mm× 64 mm. A V-notch (45°) was made in the middle of the specimen whose depth was 2.5 mm and tip of radius 0.25 mm. The specimen was placed as simply supported beam and hit by hammer from behind the notch. The impact toughness was calculated using Equation (6), where, m is hammer mass (kg), g is standard gravity (9.8 m/s^2^), h is length of hammer arm (m), β is hammer swing up angle after test piece breaks (rad), α is hammer lifting up angle (rad), w is sample width (mm), and t is sample thickness (mm).

(6) $$Impact\;toughness=\frac{mgh\left(\cos\beta -\cos\alpha \right)}{wt}$$

**Figure 2:**
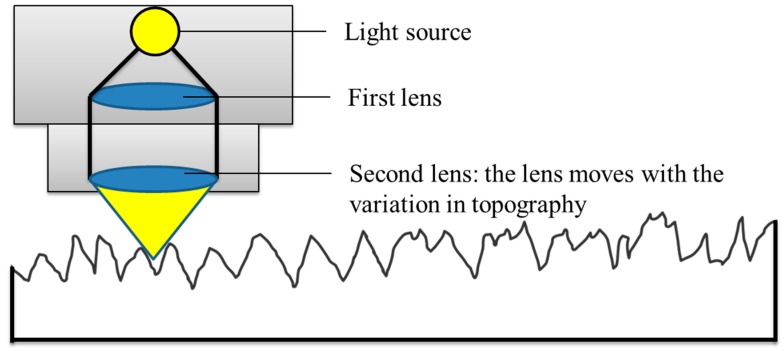
Focus-follow method for non-contact measurement of roughness.

An Alicona Infinite Focus optical microscope (G4, Alicona, Raaba/Graz, Austria) was used to generate optical micrographs and measure topographical features. The Alicona optical microscope is a non-contact method (focus-follow method) for topography measurement. The focus-follow method (Figure 2) involves the use of a moving lens which keeps a spot of light focused on the surface. The vertical movement of the lens is controlled by an electric motor (Alicona, Raaba/Graz, Austria) and correlates to the surface profile [29]. The analog electrical signal is generated to drive the motor which is then digitized and processed in the same manner as a contact stylus. A separate transducer (Alicona, Raaba/Graz, Austria) may also be used to monitor the position of the lens. Non-contact techniques are getting increasingly popular to measure topographical features, especially for surfaces that may be subject to damage using contact techniques. The results obtained are very similar to those of stylus techniques and can use the same parameter definitions. Some non-contact techniques, such as diffraction measurements, can measure topographical features easily and quickly and can potentially be used on the machining tool.

The non-contact methods have certain limitations. For example, in high slope surfaces an insufficient intensity of light reaches the detector and the focus lens begins to follow inaccurately. In addition when the contaminated surfaces are studied, the contamination is measured as part of topographical features as there is no external agency to remove the contaminations from the surface [29]. Considering these limitations, it was ensured that samples are placed flat and the surface is clean in order to obviate any artefacts in topography profiles.

DMA (Model 8000, PerkinElmer, Waltham, MA, USA) was used to determine dynamic storage modulus (*E*’) and loss modulus (*E*”) of the samples. The loss factor (Tanδ) was calculated as the ratio (*E*”/*E*’). Rectangular test specimens of dimensions 2.5 mm× 8 mm× 30 mm were used with a single cantilever clamp. All tests were carried out by temperature sweep method (temperature ramp from 30 °C to 180 °C at 5 °C/min) at a constant frequency of 1 Hz. The maximum force of DMA was 10 N and applied during all tests. The glass transition temperature (*T*_g_) was taken as the temperature value at the peak of Tanδ curves. Scanning electron microscopy analysis using a SEM FEI Quanta 200 (FEI Quanta, Hillsboro, OR, USA), was carried out of the abrasive papers to evaluate the morphology of abrasive paricles. The specimens were coated with a layer of gold using Emscope sputter coater model SC500A (Quorum technologies, Sussex, UK).

## 3. Results

**Figure 3:**
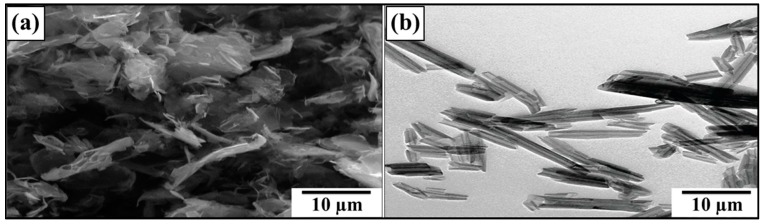
SEM images: (**a**) MLG; (**b**) nanoclay.

**Figure 4:**
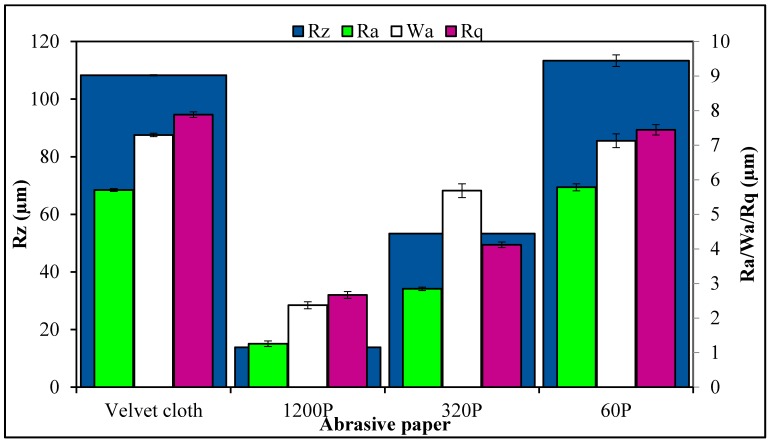
Surface roughness parameters of abrasive papers.

The SEM images of MLG and nanoclay are shown in Figure 3. Due to the wrinkled structure of MLG, stronger interfacial interactions may be expected with MLG than with the smooth tubular structure of nanoclay. The topographical features of abrasive papers are summed up in Figure 4. Surface waviness refers to the medium-frequency irregularities on the sample surface with wave-like structure comprising of series of crests and toughs [29]. Surface waviness is produced by errors in the machine tool guideway and/or the instability of the abrasive tool [29]. The waviness (*W*_a_) of velvet cloth was 1.44 µm. By treating with velvet cloth, the *W*_a_ value decreased to 1.40 µm. The *W*_a_ value of abrasive paper 1200P is 1.07 µm. The abrasive papers 320P and 60P have *W*_a_ values of 1.68 µm and 1.33 µm, respectively.

A similar trend was observed in case of surface roughness (*R*_a_). The *R*_a_ is defined as the mean height of the roughness profile and is superimposed on the surface waviness [29]. The *R*_a_ is one of the most commonly used roughness amplitude parameters. It assesses the coarseness of the surfaces, such as those produced by turning, milling and grinding operations. The *R*_a_ value of velvet cloth was 0.88 µm. The *R*_a_ value of abrasive paper 1200P was 0.71 µm. The abrasive papers 320P and 60P had *R*_a_ values of 2.84 µm and 5.78 µm, respectively.

As the averages of numbers 2 & 4 and 1 & 5 are the same, similarly, being an average value, *R*_a_ therefore cannot give accurate information about the topographical features. The disparate profiles can have the same *R*_a_ value and yet have very different performance characteristics [29]. Another average parameter, *R*_q_ states the root mean square of the profile and is more sensitive to surface variation [29]. The *R*_q_ value of velvet cloth was 1.2 µm. The *R*_q_ value of abrasive paper 1200P was 0.87 µm. The abrasive papers 320P and 60P had *R*_q_ values to 4.12 µm and 7.44 µm, respectively.

The maximum roughness height is another important parameter and was varied by treatment with abrasive papers. Sometimes, it becomes desirable to specify the maximum roughness height (*R*_max_) or peak-to-valley height (*R*_z_), rather than using *R*_a_ [29]. The *R*_z_ parameter measures the highest and lowest points of the profile and is valuable when products are subject to elevated stresses. Any large peak-to-valley heights may be areas likely to suffer from crack propagation due to stress concentration and possible triaxial state of stress generated at the notch tip [29]. However, as *R*_z_ is very susceptible to scratches or dirt, it is an unstable parameter [29]. The *R*_z_ of velvet cloth was 103.52 µm. The *R*_z_ value of abrasive paper 1200P was 12.85 µm. However, *R*_z_ values were significantly higher for abrasive papers 320P and 60P and recorded 52.32 µm and 103.46 µm respectively.

**Figure 5:**
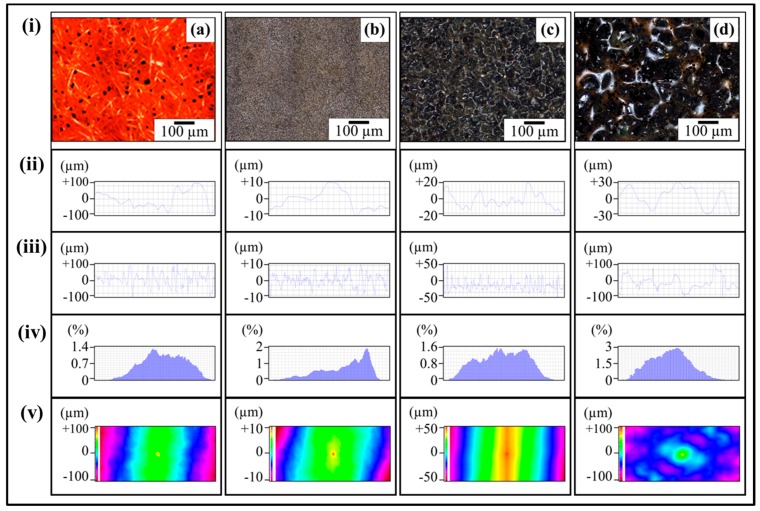
Topography profiles of abrasive papers: (**a**) Velvet cloth; (**b**) 1200P; (**c**) 320P; (**d**) 60P. In all cases; (**i**) optical image; (**ii**) waviness; (**iii**) surface roughness of selected line; (**iv**) percentage *vs.* topographical dimensions; (**v**) surface profile of selected rectangular specimen.

The topographical details of abrasive papers are shown in Figure 5. Figure 5ai shows the optical micrograph of velvet cloth where protruded fibers can be observed. Any black spots are artefacts in the optical images as these regions are either above or below the focus range. Due to the protruded fibers, the profile showed high waviness and surface roughness. The waviness of velvet cloth is shown in Figure 5aii. Although roughness amplitude is very important parameter, the spacing (waviness) of the roughness peaks can be equally important [29]. The upper bound of *W*_a_ is about ±100 µm. The *R*_a_ of velvet cloth is shown in Figure 5aiii. The surface roughness alters abruptly between ±100 µm. It is because the fibers of velvet cloth are oriented in random orientations. The Gaussian distribution of surface roughness of velvet cloth is shown in Figure 5aiv. The distribution shows typical bell-shaped curve that indicates that surface roughness is varied and most of the surface roughness values are concentrated between ±100 µm with the maximum extension up to ±120 µm. The roughness profile of complete velvet cloth sample was determined and is shown in Figure 5av. The distribution shows that *R*_z_ is about ±120 µm.

The optical micrograph of abrasive paper 1200P is shown in Figure 5bi. The abrasive particles are nearly uniformly distributed on the surface except a few rarefied regions. In addition, it was observed that the abrasive paper is not perfectly flat and contains waviness to a certain degree which is evident in Figure 5bii. The waviness varies between ±10 µm. Apart from waviness, the abrasive particles are also of non-uniform size which affects the surface profile as shown in Figure 5biii. The roughness alters sharply with distance and lies in the range of ±10 µm. The Gaussian distribution of surface roughness of abrasive paper 1200P is shown in Figure 5biv. The roughness profile of abrasive paper 1200P is shown in Figure 5bv. A major fraction of roughness is ±13 µm. As the optical micrograph (Figure 5bi) showed that 1200P is not perfectly flat, this is also evident in roughness profile (Figure 5bv).

The optical micrograph of abrasive paper 320P is shown in Figure 5ci. The abrasive powder of relatively wide size distribution is nearly uniformly distributed over the surface. The waviness (Figure 5cii) varies between ±20 µm and roughness varies between ±50 µm to −20 µm. It can also be observed that certain roughness peaks (Figure 5ciii) are pointed and other are curved that show that certain particles are sharp and other angular. Using this roughness profile, samples can be produced containing V-shaped and U-shaped notches, simultaneously. As V-shaped notches have higher associated stress concentration effect at the notch tip than that of U-shaped notches, therefore V-shaped notches will influence the mechanical properties more strongly than U-shaped notches.

The Gaussian distribution of surface roughness of abrasive paper 320P is shown in Figure 5civ. There is large size distribution and maximum concentration of roughness values reaches up to 1.6%. The roughness profile of abrasive paper 320P is shown in Figure 5cv. There is large variation of particle size with no domination of one particular size.

The optical micrograph of abrasive paper 60P is shown in Figure 5di. The abrasive particles can clearly be seen being separated by a brown phase which can be the glue to adhere the particles with the surface. As the particle size is very large, large waviness and roughness are certain. The waviness varies between ±30 µm (Figure 5dii) and roughness varies between ±100 µm (Figure 5diii). The Gaussian distribution of surface roughness is shown in Figure 5civ. A typical bell-shaped curve is obtained with ends at ±110 µm. The roughness profile of abrasive paper 60P is shown in Figure 5dv. A coarse profile is obtained due to large particle size. The *R*_z_ value goes as high as ±110 µm.

### 3.1. 0.1 wt % MLG-EP Nanocomposites

**Figure 6:**
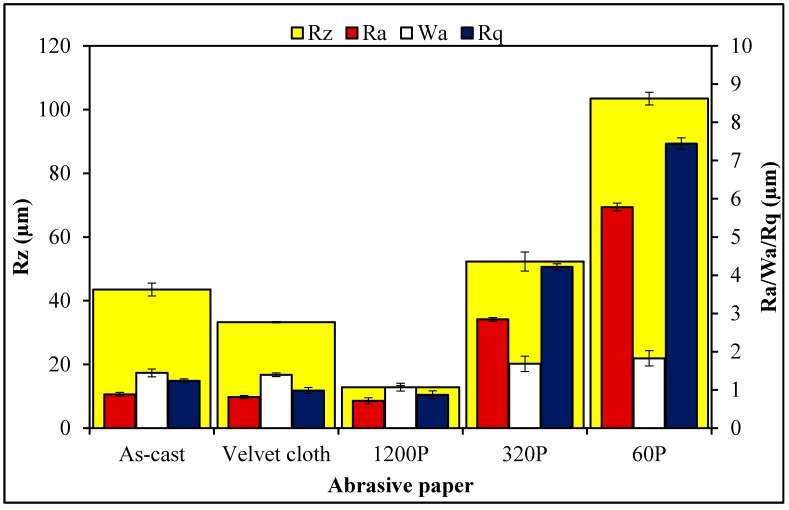
Topographical features of 0.1 wt % MLG-EP nanocomposites.

**Figure 7:**
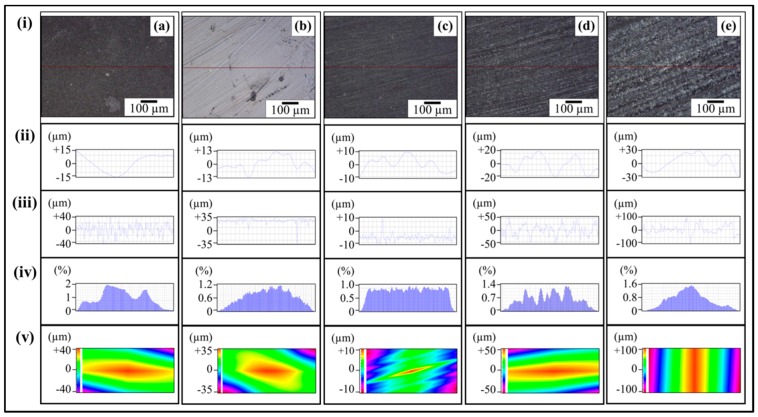
Topography profiles of 0.1 wt % MLG-EP nanocomposites treated with: (**a**) as-cast; (**b**) Velvet cloth; (**c**) 1200P; (**d**) 320P; (**e**) 60P. In all cases: (**i**) optical image; (**ii**) waviness; (**iii**) surface roughness of selected line; (**iv**) percentage *vs.* topographical dimensions; (**v**) surface profile of selected rectangular specimen.

The topographical features of 0.1 wt % MLG-EP nanocomposites are shown in Figure 6 and Figure 7. The roughness parameters were decreased by treatment with velvet cloth and 1200P while increased with 320P and 60P. Figure 7ai shows the optical micrograph of as-cast 0.1 wt % MLG-EP sample. The waviness (Figure 7aii) of the sample varies between ±15 µm while the surface roughness (Figure 7aiii) varies between ±40 µm. This surface roughness is coming from the mold surface. The surface roughness graph shows that pointed notches of about 40 µm are present on the as-cast 0.1 wt % MLG-EP samples. The Gaussian distribution (Figure 7aiv) shows that the roughness size is distributed with dominant size fraction of 2%. The roughness profile (Figure 7av) shows that most of the roughness lies within ±40 µm with a deep notch (red region).

Figure 7bi shows the optical micrograph of 0.1 wt % MLG-EP sample treated with velvet cloth for 1 min (each side) on rotating wheels with rotational speed of 150 rpm. The waviness (Figure 7bii) varies between ±13 µm while the surface roughness (Figure 7biii) varies between ±35 µm. The Gaussian distribution (Figure 7biv) shows that the roughness size is nearly uniformly distributed with dominant size fraction of 1.2%. The large range of surface roughness can be explained on the basis of diamond paste. The diamond paste had average particle size of 3 µm. Therefore, remnant dispersed and agglomerated diamond particles have contributed towards the surface roughness. The roughness profile (Figure 7bv) shows that the surface roughness slightly decreased compared to as-cast sample (Figure 7av).

Figure 7ci shows the optical micrograph of 0.1 wt % MLG-EP sample treated with abrasive paper 1200P. The waviness (Figure 7cii) varies between ±10 µm while the surface roughness (Figure 7ciii) varies between ±10 µm. The surface roughness fluctuates more quickly than in as-cast and velvet treated samples. However, the sharp notches have decreased. The Gaussian distribution (Figure 7civ) shows that a nearly uniform distribution of roughness was obtained with a dominant size fraction of 1%. The roughness profile (Figure 7cv) shows that there are no deep surface notches.

Figure 7di shows the optical micrograph of 0.1 wt % MLG-EP sample were treated with abrasive paper 320P. The scratches of different size and orientation can be observed. The waviness (Figure 7dii) varies between ±20 µm while the surface roughness (Figure 7diii) varies between ±50 µm. The Gaussian distribution (Figure 7div) shows that the dominant roughness fraction is 1.4%.

The roughness profile (Figure 7dv) shows that deep notches emerged on the sample surface by treatment with 320P.

Figure 7ei shows the optical micrograph of 0.1 wt % MLG-EP sample treated with abrasive paper 60P. A coarse topography can be observed. The waviness (Figure 7eii) varies between ±30 µm while the surface roughness (Figure 5eiii) varies between ±100 µm. The deep pointed notches can be observed which can later influence the mechanical properties of the samples. The Gaussian distribution (Figure 7eiv) shows that dominant roughness fraction is 1.6%. The surface profile of larger sample (Figure 7ev) shows that coarse topography is present with abruptly changing roughness.

**Figure 8:**
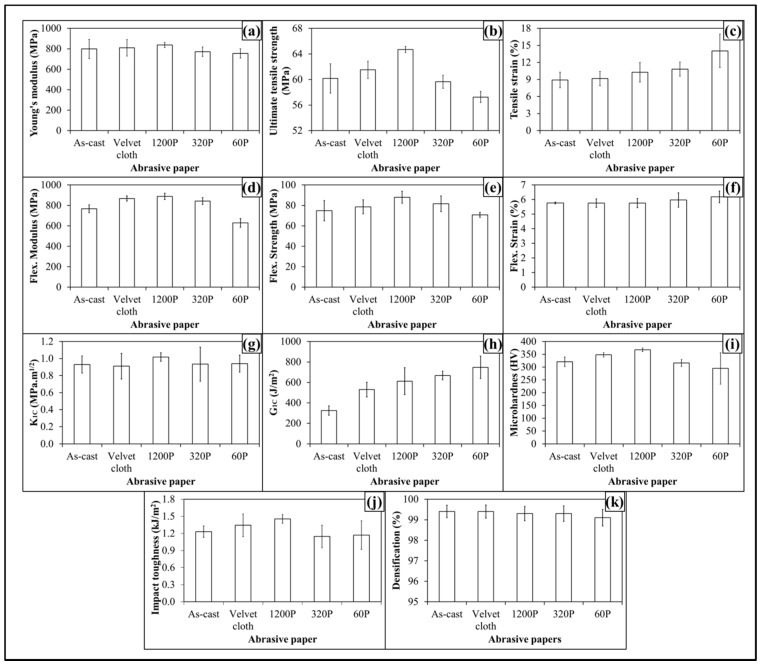
Mechanical properties of 0.1 wt % MLG-EP nanocomposites: (**a**) Young’s modulus (MPa); (**b**) UTS (MPa); (**c**) tensile strain (%); (**d**) flexural modulus (MPa); (**e**) flexural strength (MPa); (**f**) flexural strain (%); (**g**) K_1C_ (MPa.m^1/2^); (**h**) G_1C_ (J/m^2^); (**i**) microhardness (HV); (**j**) Charpy impact toughness (kJ/m^2^); (**k**) densification (%).

The topographical features influenced mechanical properties of 0.1 wt % MLG-EP samples as shown in Figure 8. The Young’s modulus (Figure 8a) increased from 798 MPa to 810 MPa (1.4% increase) when 0.1 wt % MLG-EP nanocomposites were treated with velvet cloth. The Young’s modulus of 0.1 wt % MLG-EP nanocomposites treated with 1200P also increased to 838 MPa (5% increase). However the Young’s modulus of 0.1 wt % MLG-EP nanocomposites treated with 320P decreased to 772 MPa (3.3% decrease). The maximum decrease in Young’s modulus was observed when the samples were treated with abrasive paper 60P and decreased to 755 MPa (5.5% decrease). The values show that Young’s modulus can be increased by treatment with velvet cloth and abrasive paper 1200P and decreased by treatment with abrasive papers 320P and 60P.

The influence of topography on UTS is shown in Figure 8b. The UTS of 0.1 wt % MLG-EP nanocomposites treated with velvet cloth increased from 60 MPa to 62 MPa (2.2% increase). The maximum increase in UTS of 0.1 wt % MLG-EP nanocomposites was observed when 0.1 wt % MLG-EP nanocomposites were treated with abrasive paper 1200P and UTS increased to 65 MPa (7.5% increase). The UTS of 0.1 wt % MLG-EP nanocomposites treated with abrasive paper 320P decreased to 59 MPa (1% decrease). When treated with abrasive papers 60P, the UTS decreased to 57 MPa (4.8% decrease). The influence of topography on tensile strain is shown in Figure 8c. The tensile strain kept increasing with the coarser topography which can be attributed to lower stiffness and strength values. The tensile strain did not change much with velvet cloth and slightly increased in the case of abrasive paper 1200P. Therefore, overall better tensile properties can be achieved when samples are treated with velvet cloth and abrasive paper 1200P.

The influence of topography on flexural modulus of 0.1 wt % MLG-EP nanocomposites is shown in Figure 8d. The flexural modulus of as-cast 0.1 wt % MLG-EP nanocomposites is 767 MPa. When treated with velvet cloth, the flexural modulus increases to 867 MPa (13% increase). The flexural modulus of 0.1 wt % MLG-EP nanocomposites treated with abrasive paper 1200P is 888 MPa (16% increase). The flexural modulus of sample treated with 320P is 842 MPa (10% increase) and that of treated with 60P is 629 MPa which shows a decrease of 18%. The variation in flexural modulus is nearly in accordance with the variation in tensile modulus.

The influence of topography on flexural strength is shown in Figure 8e. The flexural strength of as-cast 0.1 wt % MLG-EP nanocomposites is 75 MPa. When treated with velvet cloth, the flexural strength increases to 79 MPa (5% increase). The flexural strength of sample treated with abrasive paper 1200P is 88 MPa (18% increase). The flexural strength of sample treated with 320P remained high and was 82 MPa (9% increase). In comparison to tensile strength which increased only to 3%, an increase of 22% in flexural strength indicates that surface roughness up to ±20 µm is not detrimental to flexural strength. The flexural strength of sample treated with abrasive paper 60P is 70 MPa which is slightly lower than that of as-cast sample. In comparison to tensile strength which decreased by 2%, the flexural strength value of sample treated with 60P indicates that surface roughness up to ±30 µm has detrimental effect on tensile strength while flexural strength seems impervious. The influence of topography on flexural strain (%) is shown in Figure 8f. The flexural strain nearly remained the same till abrasive paper 320P. However, it increased when sample was treated with abrasive paper 60P. This increase in flexural strain can be explained on the basis of decreased flexural modulus and strength.

The influence of topography on fracture toughness (*K*_1C_) is shown in Figure 8g. No specific trend on *K*_1C_ was observed and *K*_1C_ remained nearly the same. The values show that standard deviation is different for different samples. This can be because the tip of the notch was sharpened manually with a fresh razor blade which may not generate surfaces of equal length and curvature. In addition, the volume fraction, size, and distribution of porosity can be another factor which can influence the mechanical properties. The variation in *G*_1C_ of topographically modified 0.1 wt % MLG-EP samples is shown in Figure 8h. The trend shows that *G*_1C_ increases as the coarseness of topography increases. However, as topography did not show significant influence on *K*_1C_, the authors are skeptical in believing that this increase in *G*_1C_ is directly coming from topography. In calculating *G*_1C_, $K_{1C}^{2}$ is divided by Young’s modulus. As Young’s modulus decreased with coarse topography, therefore the increase in G_1C_ is possibly stemming from decreased Young’s modulus.

The variation in microhardness is shown in Figure 8i. The hardness of as-cast sample is 321 HV. When treated with velvet cloth, the microhardness increased to 348 HV (8% increase). When treated with abrasive paper 1200P, microhardness increases to 368 HV (14% increase). The microhardness values of samples treated with abrasive papers 320P and 60 were 316 HV (3% decrease) and 265 HV (5% decrease).

The Charpy impact toughness values are shown in Figure 8j. The Charpy impact toughness value of as-cast 0.1 wt % MLG-EP nanocomposites is 1.2 kJ/m^2^. After treatment with velvet cloth, the Charpy impact toughness increased to 1.3 kJ/m^2^ (9% increase). The values of samples treated with abrasive papers 1200P, 320P and 60P are 1.5, 1.2, and 1.1 kJ/m^2^, respectively. Although there was no significant difference observed in fracture toughness values, however, treatment of 0.1 wt % MLG-EP samples with abrasive papers showed a significant impact on Charpy impact toughness values.

The densification of 0.1 wt % MLG-EP samples is shown in Figure 8k. The densification of as-cast 0.1 wt % MLG-EP samples is 99.5% (mean value). The densification slightly decreased when the samples were treated with abrasive papers. The mean values of densification were 99.4%, 99.4%, 99.2%, and 99.1%, when treated with velvet cloth, 1200P, 320P, and 60P, respectively. This variation in densification may be either caused by the treatment with abrasive papers or the samples already had the same densification values as the casting technique is not usually considered 100% reproducible like latex technology [39].

**Figure 9:**
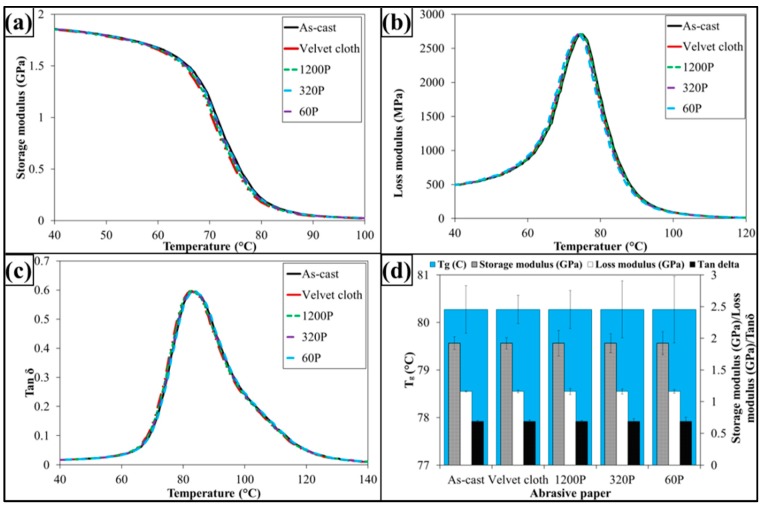
Dynamic mechanical properties of 0.1 wt % MLG-EP nanocomposites: (**a**) Storage modulus; (**b**) Loss modulus; (**c**) Loss factor (Tanδ); (**d**) Glass transition temperature (*T*_g_), storage modulus, loss modulus and Tanδ values corresponding to *T*_g_.

The dynamic mechanical properties are shown in Figure 9. The treatment with abrasive papers has not influenced dynamic mechanical properties of 0.1 wt % MLG-EP samples. This is because the dynamic mechanical properties are mainly dependent on the structure of the sample. As treatment with abrasive papers did not change structure of the nanocomposites, therefore it did not change the dynamic mechanical properties. It can further be concluded that surface notches up to ±100 µm do not change the dynamic mechanical properties of nanocomposites.

### 3.2. 0.1 wt % Clay-EP Nanocomposites

**Figure 10:**
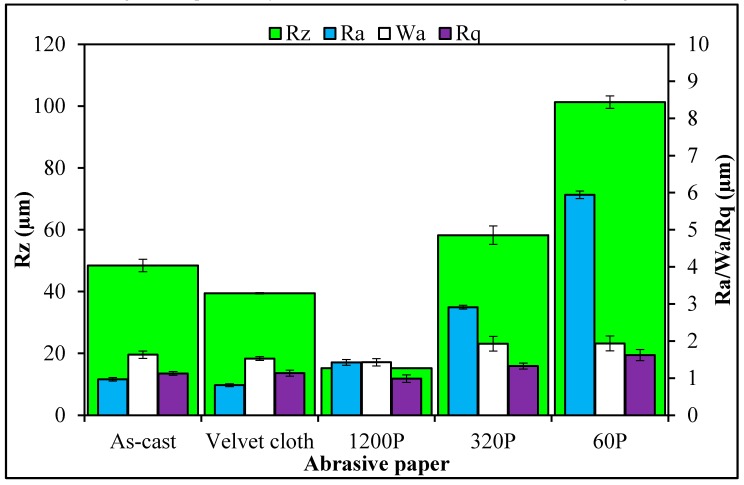
Topographical features of 0.1 wt % clay-EP nanocomposites.

**Figure 11:**
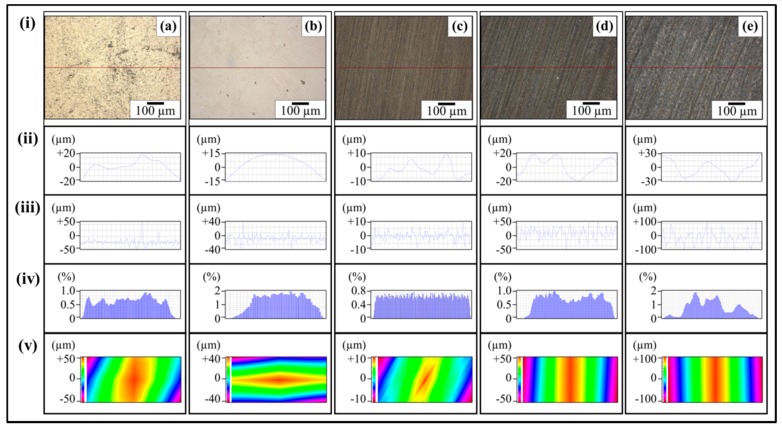
Topography profiles of 0.1 wt % clay-EP nanocomposites treated with: (**a**) As-cast; (**b**) Velvet cloth; (**c**) 1200P; (**d**) 320P; (**e**) 60P. In all cases: (**i**) Optical image; (**ii**) Waviness; (**iii**) Surface roughness of selected line; (**iv**) Percentage *vs.* topographical dimensions; (**v**) Surface profile of selected rectangular specimen.

The topographical features of 0.1 wt % clay-EP nanocomposites are shown in Figure 10 and details shown in Figure 11. The roughness parameters were decreased by treatment with velvet cloth and 1200P while increased with 320P and 60P. Figure 11ai shows the optical micrograph of as-cast 0.1 wt % clay-EP sample. The waviness (Figure 11aii) of the sample varies between ±15 µm while the surface roughness (Figure 11aiii) varies between ±40 µm. This surface roughness is coming from the mold surface. The surface roughness graph shows that pointed notches of about 40 µm are present on the as-cast 0.1 wt % clay-EP samples. The Gaussian distribution (Figure 11aiv) shows that the roughness size is distributed with dominant size fraction of 1%. The roughness profile (Figure 11av) shows that most of the roughness lies within ±40 µm with a deep notch (red region).

Figure 11bi shows the optical micrograph of 0.1 wt % clay-EP sample treated with velvet cloth for 1 min (each side) on rotating wheels with a rotational speed of 150 rpm. The waviness (Figure 11bii) varies between ±15 µm while the surface roughness (Figure 11biii) varies between ±35 µm. The Gaussian distribution (Figure 11biv) shows that the roughness size is nearly uniformly distributed with a dominant size fraction of 2%. The large range of surface roughness can be explained on the basis of diamond paste. The diamond paste had an average particle size of 3 µm. Therefore, remnant dispersed and agglomerated diamond particles contributed toward surface roughness. The roughness profile (Figure 11bv) shows that the surface roughness slightly decreased compared to as-cast sample (Figure 11av). Figure 11ci shows the optical micrograph of 0.1 wt % clay-EP sample treated with abrasive paper 1200P. The waviness (Figure 11cii) varies between ±10 µm while the surface roughness (Figure 11ciii) also varies between ±10 µm. The surface roughness fluctuates more quickly than in as-cast and velvet treated samples. However, the sharp notches have decreased. The Gaussian distribution (Figure 11civ) shows that a nearly uniform distribution of roughness was obtained with a dominant size fraction of 0.8%. The roughness profile (Figure 11cv) shows that there are no deep surface notches.

Figure 11di shows that the optical micrograph of 0.1 wt % clay-EP sample treated with abrasive paper 320P. The scratches of different size and orientation can be observed. The waviness (Figure 11dii) varies between ±20 µm while the surface roughness (Figure 11diii) varies between ±50 µm. The Gaussian distribution (Figure 11div) shows that the dominant roughness fraction is 1%. The roughness profile (Figure 11dv) shows that deep notches emerge on the sample surface by treatment with 320P.

Figure 11ei shows the optical micrograph of 0.1 wt % clay-EP sample treated with abrasive paper 60P. A coarse topography can be observed. The waviness (Figure 11eii) varies between ±30 µm while the surface roughness (Figure 11eiii) varies between ±100 µm. The deep pointed notches can be observed which can later influence the mechanical properties of the samples. The Gaussian distribution (Figure 11eiv) shows that the dominant roughness fraction is 2%. The surface profile of the larger sample (Figure 11ev) shows that coarse topography is present with abruptly changing roughness.

**Figure 12:**
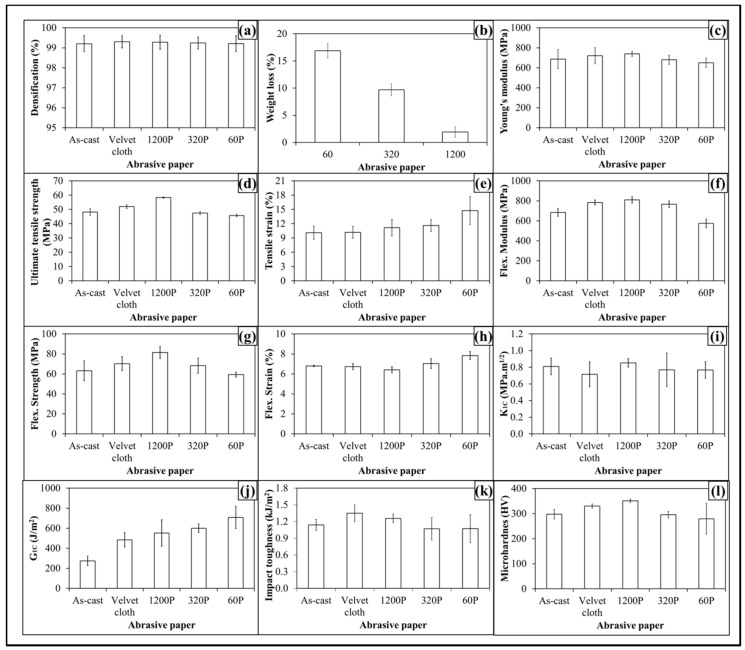
(**a**) Densification; (**b**) Weight loss (%); (**c**–**l**) Mechanical properties of 0.1 wt % clay-EP nanocomposites; (**c**) Young’s modulus (MPa); (**d**) UTS (MPa); (**e**) Tensile strain (%); (**f**) Flexural modulus (MPa); (**g**) Flexural strength (MPa); (**h**) Flexural strain (%); (**i**) *K*_1C_ (MPa.m^1/2^); (**j**) *G*_1C_ (J/m^2^); (**k**) Charpy impact toughness (kJ/m^2^); (**l**) Microhardness (HV).

The produced nanocomposites had a densification of about 99.5% (Figure 12a) and weight loss of about 16% (Figure 12b) when samples were treated with abrasive paper 60P. The topographical features influenced the mechanical properties of 0.1 wt % clay-EP samples as shown in Figure 12(c–l). The Young’s modulus (Figure 12c) increased from 687 MPa to 720 MPa (4.8% increase) when 0.1 wt % clay-EP nanocomposites was treated with velvet cloth. The Young’s modulus of 0.1 wt % clay-EP nanocomposites treated with 1200P also increased to 740 MPa (7.6% increase). However, the Young’s modulus of 0.1 wt % clay-EP nanocomposites treated with 320P decreased to 681 MPa (1% decrease). The maximum decrease in Young’s modulus was observed when the samples were treated with abrasive paper 60P and decreased to 650 MPa (5.8% decrease). The values show that Young’s modulus can be increased by treatment with velvet cloth and abrasive paper 1200P and decreased by treatment with abrasive papers 320P and 60P.

The influence of topography on UTS is shown in Figure 12d. The UTS of 0.1 wt % clay-EP nanocomposites treated with velvet cloth increased from 48 MPa to 52 MPa (8% increase). The maximum increase in UTS of 0.1 wt % clay-EP nanocomposites was observed when 0.1 wt % clay-EP nanocomposites treated with abrasive paper 1200P and UTS increased to 58 MPa (20% increase). The UTS of 0.1 wt % clay-EP nanocomposites treated with abrasive paper 320P decreased to 47 MPa (1.5% decrease). When treated with abrasive papers 60P, the UTS decreased to 45.8 MPa (5% decrease). The influence of topography on tensile strain is shown in Figure 12e. The tensile strain kept increasing with the coarser topography which can be attributed to lower stiffness and strength values. The tensile strain did not change much with velvet cloth and slightly increased in the case of abrasive paper 1200P. Therefore, overall better tensile properties can be achieved when samples are treated with velvet cloth and abrasive paper 1200P.

The influence of topography on flexural modulus of 0.1 wt % clay-EP nanocomposites is shown in Figure 12f. The flexural modulus of as-cast 0.1 wt % clay-EP nanocomposites is 684 MPa. When treated with velvet cloth, the flexural modulus increased to 783 MPa (15% increase). The flexural modulus of 0.1 wt % clay-EP nanocomposites treated with abrasive paper 1200P is 810 MPa (18% increase). The flexural modulus of the sample treated with 320P is 766 MPa (12% increase) and that treated with 60P is 575 MPa which shows a decrease of 16%. The variation in flexural modulus is nearly in accordance with the variation in tensile modulus.

The influence of topography on flexural strength is shown in Figure 12g. The flexural strength of as-cast 0.1 wt % clay-EP nanocomposites is 63 MPa. When treated with velvet cloth, the flexural strength increases to 70 MPa (11% increase). The flexural strength of the sample treated with abrasive paper 1200P is 81 MPa (29% increase). The flexural strength of the sample treated with 320P remained high and was 68 MPa (8% increase). In comparison to tensile strength which increased only to 3%, an increase of 29% in flexural strength indicates that surface roughness up to ±20 µm is not detrimental to flexural strength. The flexural strength of a sample treated with abrasive paper 60P is 59 MPa which is slightly lower than that of as-cast sample. In comparison to the tensile strength which decreased by 6%, the flexural strength value of the sample treated with 60P indicates that the surface roughness up to ±30 µm has a detrimental effect on tensile strength, while flexural strength seems impervious. The influence of topography on flexural strain (%) is shown in Figure 12h. The flexural strain nearly remained the same till abrasive paper 320P was used. However, it increased when the sample was treated with abrasive paper 60P. This increase in flexural strain can be explained on the basis of decreased flexural modulus and strength.

The influence of topography on fracture toughness (*K*_1C_) is shown in Figure 12i. No specific trend on *K*_1C_ was observed and *K*_1C_ remained nearly the same. The values show that standard deviation is different for different samples. It can be because the tip of notch was sharpened manually by a fresh razor blade which may not generate surfaces of equal length and curvature. In addition, the volume fraction, size and distribution of porosity can be another factor which can influence the mechanical properties. The variation in *G*_1C_ of topographically modified 0.1 wt % clay-EP samples is shown in Figure 12j. The trend shows that *G*_1C_ increases as the coarseness of topography increases. However, as topography did not show significant influence on *K*_1C_, the authors are skeptical in believing that this increase in *G*_1C_ is directly coming from topography. In calculating *G*_1C_, $K_{1C}^{2}$ is divided by Young’s modulus. As Young’s modulus decreased with coarse topography, therefore the increase in *G*_1C_ is possibly stemming from decreased Young’s modulus.

The Charpy impact toughness values are shown in Figure 12k. The Charpy impact toughness value of as-cast 0.1 wt % clay-EP nanocomposites is 1.2 kJ/m^2^. After treatment with velvet cloth, the Charpy impact toughness increased to 1.3 kJ/m^2^ (9% increase). The values of samples treated with abrasive papers 1200P, 320P and 60P are 1.5, 1.2, and 1.1 kJ/m^2^, respectively. Although there was no significant difference observed in fracture toughness values, however treatment of 0.1 wt % clay-EP samples with abrasive papers showed a significant impact on Charpy impact toughness values.

The variation in microhardness is shown in Figure 12l. The hardness of as-cast sample is 297 HV. When treated with velvet cloth, the microhardness increased to 329 HV (11% increase). When treated with abrasive paper 1200P, microhardness increased to 351 HV (18% increase). The microhardness values of samples treated with abrasive papers 320P and 60P were 296 HV (1% decrease) and 279 HV (6% decrease).

**Figure 13:**
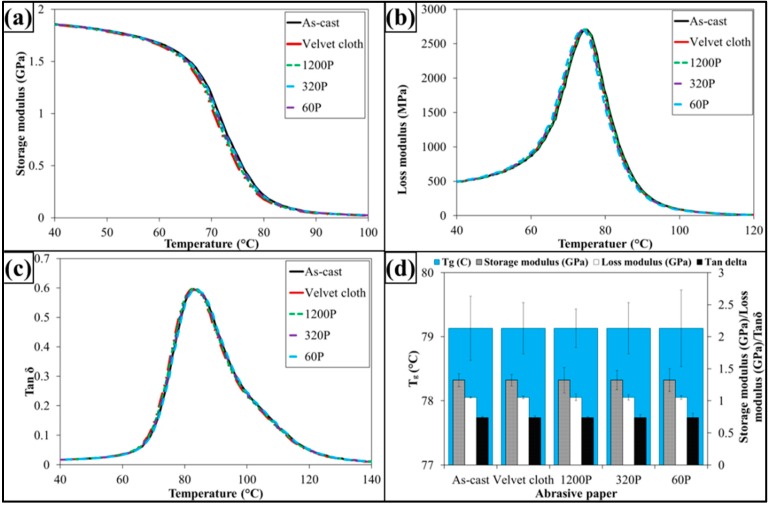
Dynamic mechanical properties of 0.1 wt % clay-EP nanocomposites: (**a**) Storage modulus; (**b**) Loss modulus; (**c**) Loss factor (Tanδ); (**d**) Glass transition temperature (*T*_g_), Storage modulus, loss modulus and Tanδ values corresponding to *T*_g_.

The dynamic mechanical properties are shown in Figure 13. The treatment with abrasive papers has not influenced dynamic mechanical properties of 0.1 wt % clay-EP samples. It is because the dynamic mechanical properties are mainly dependent on the structure of sample. As treatment with abrasive papers did not change structure of the nanocomposites, therefore it did not change the dynamic mechanical properties. It can further be concluded that surface notches up to ±100 µm do not change the dynamic mechanical properties of nanocomposites.

### 3.3. 0.05 wt % MLG-0.05 wt % Clay-EP Nanocomposites

**Figure 14:**
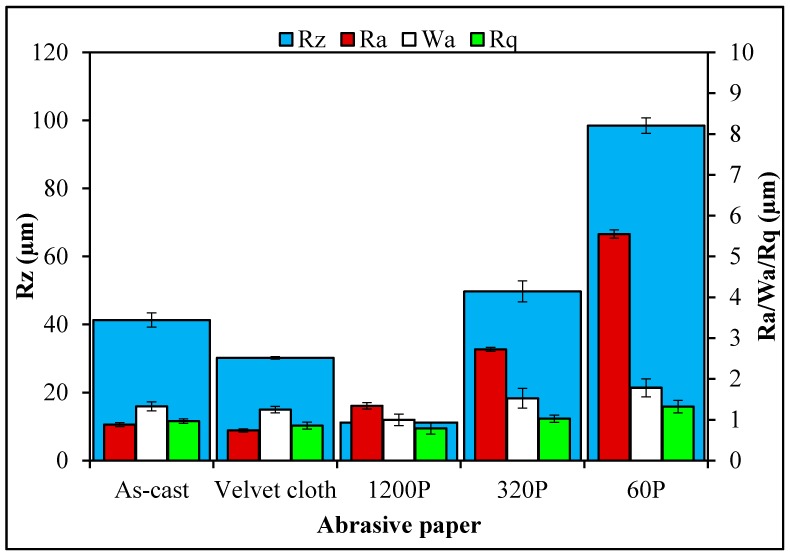
Topographical features of 0.05 wt % MLG-0.05 wt % clay-EP nanocomposites.

**Figure 15:**
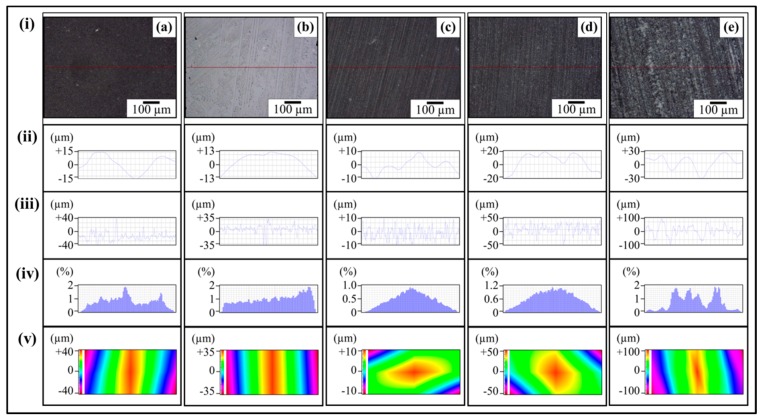
Topography profiles of 0.05 wt % MLG-0.05 wt % clay-EP samples: (**a**) As-cast; (**b**) Velvet cloth; (**c**) 1200P; (**d**) 320P; (**e**) 60P. In all the cases: (**i**) optical image; (**ii**) waviness; (**iii**) surface roughness of selected line; (**iv**) percentage *vs.* topographical dimensions; (**v**) surface profile of selected rectangular specimen.

The topographical features of 0.05 wt % MLG-0.05 wt % clay-EP nanocomposites are shown in Figure 14 and details shown in Figure 15. The roughness parameters were decreased by treatment with velvet cloth and 1200P, while increased with 320P and 60P. Figure 15ai shows the optical micrograph of as-cast 0.05 wt % MLG-0.05 wt % clay-EP sample. The waviness (Figure 15aii) of the sample varies between ±15 µm while the surface roughness (Figure 15aiii) varies between ±40 µm. This surface roughness is coming from the mold surface. The surface roughness graph shows that pointed notches of about 40 µm are present on the as-cast 0.05 wt % MLG-0.05 wt % clay-EP samples. The Gaussian distribution (Figure 15aiv) shows that the roughness size is distributed with dominant size fraction of 1%. The roughness profile (Figure 15av) shows that most of the roughness lies within ±40 µm with a deep notch (red region).

Figure 15bi shows the optical micrograph of 0.05 wt % MLG-0.05 wt % clay-EP sample treated with velvet cloth for 1 min (each side) on rotating wheels with rotational speed of 150 rpm. The waviness (Figure 15bii) varies between ±15 µm while the surface roughness (Figure 15biii) varies between ±35 µm. The Gaussian distribution (Figure 15biv) shows that the roughness size is nearly uniformly distributed with dominant size fraction of 2%. The large range of surface roughness can be explained on the basis of diamond paste. The diamond paste had average particle size of 3 µm. Therefore, remnant dispersed and agglomerated diamond particles contributed toward surface roughness. The roughness profile (Figure 15bv) shows that the surface roughness slightly decreased compared to as-cast sample (Figure 15av).

Figure 15ci shows the optical micrograph of 0.05 wt % MLG-0.05 wt % clay-EP sample treated with abrasive paper 1200P. The waviness (Figure 15cii) varies between ±10 µm while the surface roughness (Figure 15ciii) varies between ±12 µm. The surface roughness fluctuates more quickly than in as-cast and velvet treated samples. However, the sharp notches have decreased. The Gaussian distribution (Figure 15civ) shows that a nearly uniform distribution of roughness was obtained with dominant size fraction of 0.8%. The roughness profile (Figure 15cv) shows that there are no deep surface notches.

Figure 15di shows that the optical micrograph of 0.05 wt % MLG-0.05 wt % clay-EP sample treated with abrasive paper 320P. The scratches of different size and orientation can be observed. The waviness (Figure 15dii) varies between ±20 µm while the surface roughness (Figure 15diii) varies between ±50 µm. The Gaussian distribution (Figure 15div) shows that the dominant roughness fraction is 1%. The roughness profile (Figure 15dv) shows that deep notches emerge on sample surface by treatment with 320P.

Figure 15ei shows the optical micrograph of 0.05 wt % MLG-0.05 wt % clay-EP sample treated with abrasive paper 60P. A coarse topography can be observed. The waviness (Figure 15eii) varies between ±30 µm while the surface roughness (Figure 15eiii) varies between ±100 µm. The deep pointed notches can be observed which can later influence the mechanical properties of the samples. The Gaussian distribution (Figure 15eiv) shows that dominant roughness fraction is 2%. The surface profile of a larger sample (Figure 15ev) shows that coarse topography is present with abruptly changing roughness.

**Figure 16:**
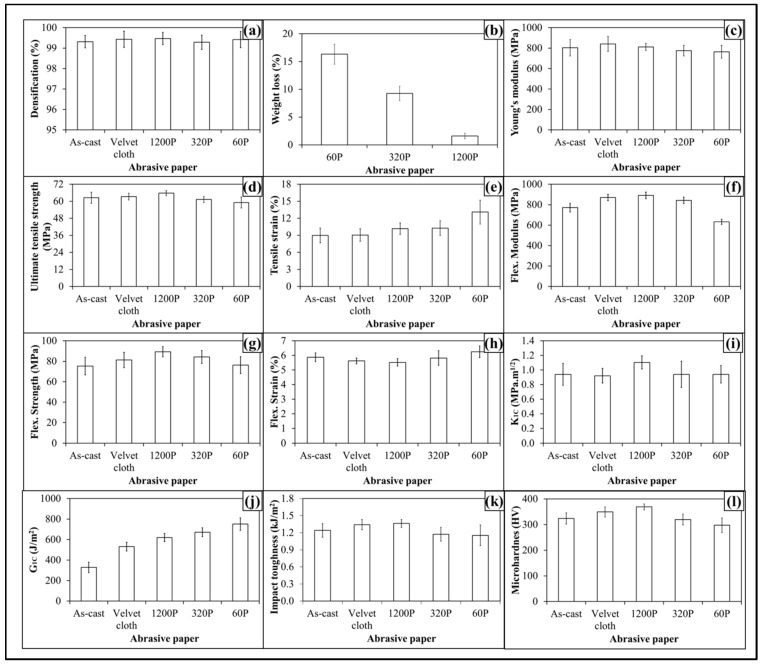
(**a**) Densification; (**b**) weight loss (%); (**c**–**l**) Mechanical properties of 0.05 wt % MLG-0.05 wt % clay-EP nanocomposites: (**c**) Young’s modulus (MPa); (**d**) UTS (MPa); (**e**) tensile strain (%); (**f**) flexural modulus (MPa); (**g**) flexural strength (MPa); (**h**) flexural strain (%); (**i**) *K*_1C_ (MPa.m^1/2^); (**j**) *G*_1C_ (J/m^2^); (**k**) Charpy impact toughness (kJ/m^2^); (**l**) Microhardness (HV).

The produced nanocomposites had densification of about 99.5% (Figure 16a) and weight loss of about 16% (Figure 16b) when samples were treated with abrasive paper 60P. The topographical features influenced mechanical properties of 0.05 wt % MLG-0.05 wt % clay-EP samples as shown in Figure 16(c–l). The Young’s modulus (Figure 16c) increased from 687 MPa to 720 MPa (4.8% increase) when 0.05 wt % MLG-0.05 wt % clay-EP nanocomposites were treated with velvet cloth. The Young’s modulus of 0.05 wt % MLG-0.05 wt % clay-EP nanocomposites treated with 1200P also increased to 740 MPa (7.6% increase). However, the Young’s modulus of 0.05 wt % MLG-0.05 wt % clay-EP nanocomposites treated with 320P decreased to 681 MPa (1% decrease). The maximum decrease in Young’s modulus was observed when the samples were treated with abrasive paper 60P and decreased to 650 MPa (5.8% decrease). The values show that Young’s modulus can be increased by treatment with velvet cloth and abrasive paper 1200P and decreased by treatment with abrasive papers 320P and 60P.

The influence of topography on UTS is shown in Figure 16d. The UTS of 0.05 wt % MLG-0.05 wt % clay-EP nanocomposites treated with velvet cloth increased from 48 MPa to 52 MPa (8% increase). The maximum increase in UTS of 0.05 wt % MLG-0.05 wt % clay-EP nanocomposites was observed when 0.05 wt % MLG-0.05 wt % clay-EP nanocomposites were treated with abrasive paper 1200P and UTS increased to 58 MPa (20% increase). The UTS of 0.05 wt % MLG-0.05 wt % clay-EP nanocomposites treated with abrasive paper 320P decreased to 47 MPa (1.5% decrease). When treated with abrasive papers 60P, the UTS decreased to 45.8 MPa (5% decrease). The influence of topography on tensile strain is shown in Figure 16e. The tensile strain kept increasing with coarser topography which can be attributed to lower stiffness and strength values. The tensile strain did not change much with velvet cloth and slightly increased in the case of abrasive paper 1200P. Therefore, overall better tensile properties can be achieved when samples are treated with velvet cloth and abrasive paper 1200P.

The influence of topography on flexural modulus of 0.05 wt % MLG-0.05 wt % clay-EP nanocomposites is shown in Figure 16f. The flexural modulus of as-cast 0.05 wt % MLG-0.05 wt % clay-EP nanocomposites is 684 MPa. When treated with velvet cloth, the flexural modulus increased to 783 MPa (15% increase). The flexural modulus of 0.05 wt % MLG-0.05 wt % clay-EP nanocomposites treated with abrasive paper 1200P is 810 MPa (18% increase). The flexural modulus of the sample treated with 320P is 766 MPa (12% increase) and that treated with 60P is 575 MPa which shows a decrease of 16%. The variation in flexural modulus is nearly in accordance with the variation in tensile modulus.

The influence of topography on flexural strength is shown in Figure 16g. The flexural strength of as-cast 0.05 wt % MLG-0.05 wt % clay-EP nanocomposites is 63 MPa. When treated with velvet cloth, the flexural strength increased to 70 MPa (11% increase). The flexural strength of sample treated with abrasive paper 1200P was 81 MPa (29% increase). The flexural strength of sample treated with 320P remained high and was 68 MPa (8% increase). In comparison to tensile strength which increased only to 3%, an increase of 29% in flexural strength indicates that surface roughness up to ±20 µm is not detrimental to flexural strength. The flexural strength of sample treated with abrasive paper 60P is 59 MPa which is slightly lower than that of as-cast sample. In comparison to tensile strength which decreased by 6%, the flexural strength value of sample treated with 60P indicates that surface roughness up to ±30 µm has detrimental effect on tensile strength while flexural strength seems impervious. The influence of topography on flexural strain (%) is shown in Figure 16h. The flexural strain nearly remained the same till abrasive paper 320P. However, it increased when sample was treated with abrasive paper 60P. This increase in flexural strain can be explained on the basis of decreased flexural modulus and strength.

The influence of topography on fracture toughness (*K*_1C_) is shown in Figure 16i. No specific trend on *K*_1C_ was observed and *K*_1C_ remained nearly the same. The values show that a standard deviation is different for different samples. It can be because the tip of notch was sharpened manually by a fresh razor blade which may not generate surfaces of equal length and curvature. In addition, the volume fraction, size and distribution of porosity can be another factor which can influence the mechanical properties. The variation in *G*_1C_ of topographically modified 0.05 wt % MLG-0.05 wt % clay-EP samples is shown Figure 16j. The trend shows that *G*_1C_ increases as the coarseness of topography increases. However, as topography did not show significant influence on *K*_1C_, the authors are skeptical in believing that this increase in *G*_1C_ is directly coming from topography. In calculating *G*_1C_, $K_{1C}^{2}$ is divided by Young’s modulus. As Young’s modulus decreased with coarse topography, therefore the increase in *G*_1C_ is possibly stemming from decreased Young’s modulus.

The Charpy impact toughness values are shown in Figure 16j. The Charpy impact toughness value of as-cast 0.05 wt % MLG-0.05 wt % clay-EP nanocomposites is 1.2 kJ/m^2^. After treatment with velvet cloth, the 0.05 wt % MLG-0.05 wt % clay-EP nanocomposites increased to 1.3 kJ/m^2^ (9% increase). The values of samples treated with abrasive papers 1200P, 320P and 60P are 1.5, 1.2 and 1.1 kJ/m^2^, respectively. Although there was no significant difference observed in fracture toughness values, however treatment of 0.05 wt % MLG-0.05 wt % clay-EP samples with abrasive papers showed a significant impact on Charpy impact toughness values.

The variation in microhardness is shown in Figure 16l. The hardness of as-cast sample is 297 HV. When treated with velvet cloth, the microhardness increased to 329 HV (11% increase). When treated with abrasive paper 1200P, microhardness increased to 351 HV (18% increase). The microhardness values of samples treated with abrasive papers 320P and 60P were 296 HV (1% decrease) and 279 HV (6% decrease).

**Figure 17:**
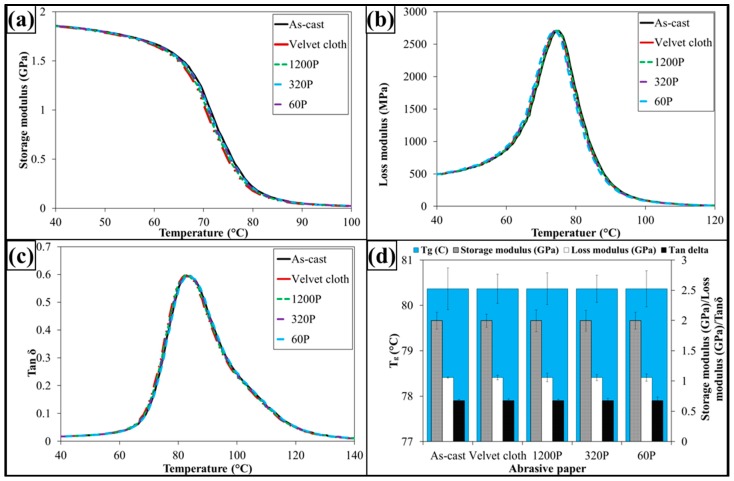
Dynamic mechanical properties of 0.05 wt % MLG-0.05 wt % clay-EP nanocomposites: (**a**) storage modulus; (**b**) loss modulus; (**c**) loss factor (Tanδ); (**d**) glass transition temperature (*T*_g_), storage modulus, loss modulus and Tanδ values corresponding to *T*_g_.

The dynamic mechanical properties are shown in Figure 17. The treatment with abrasive papers has not influenced dynamic mechanical properties of 0.05 wt % MLG-0.05 wt % clay-EP samples. It is because the dynamic mechanical properties are mainly dependent on the structure of the sample. As treatment with abrasive papers did not change the structure of the nanocomposites, therefore it did not change the dynamic mechanical properties. It can further be concluded that surface notches up to ±100 µm do not change the dynamic mechanical properties of nanocomposites.

**Figure 18:**
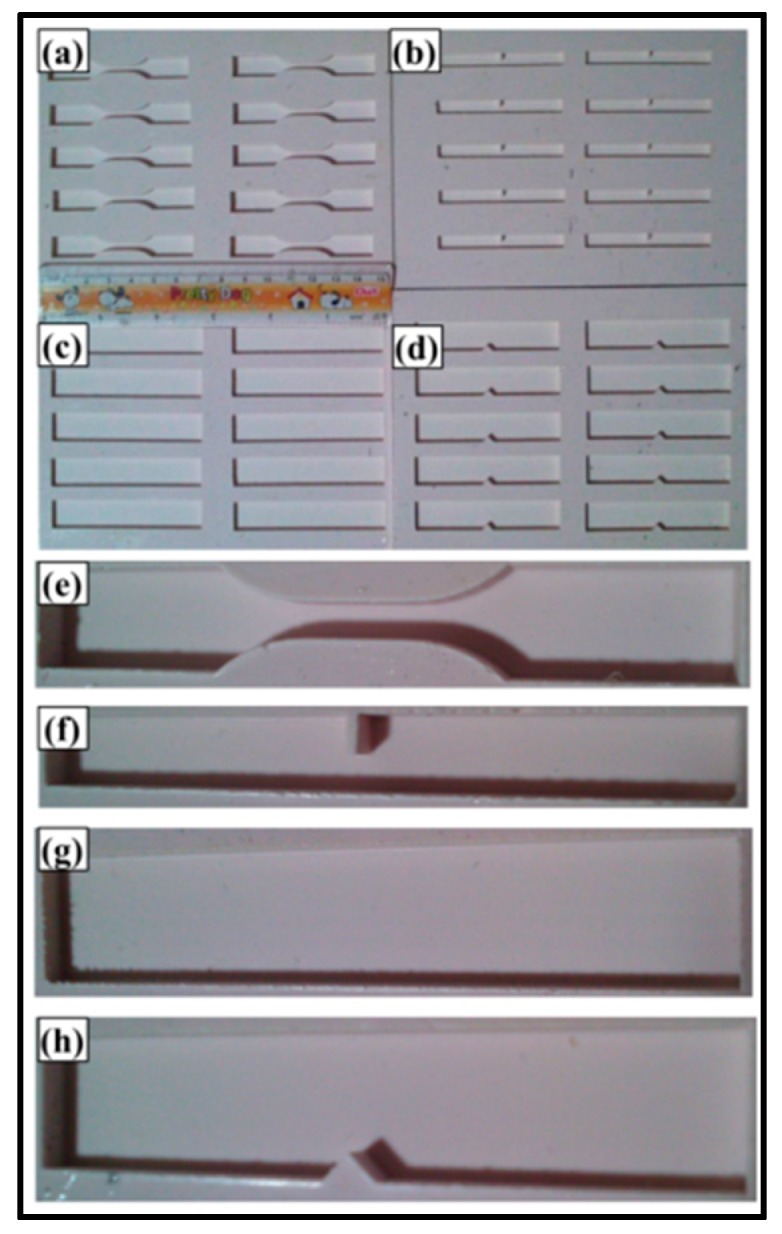
Mold photographs: (**a**) Tensile; (**b**) *K*_1C_; (**c**) Flexural; (**d**) Charpy impact toughness molds; magnified images shown in (**e**–**h**) respectively.

It can be observed that as-cast samples had surface notches stemming from the mold surface as shown in Figure 18. The surface notches in as-cast samples seem inevitable as vibrations and possible wobbling of machine tool yields notches on the surface of the mold the impression of which is transferred to the surface of samples cast.

The increase in Young’s modulus and UTS with velvet cloth and abrasive paper 1200P can be attributed to the smoothening of the surfaces as as-cast specimens had surface roughness values between ±43 µm. In comparison, the surface roughness of 0.1 wt % MLG/clay-EP nanocomposites treated with velvet cloth varied between ±33 µm and that of abrasive paper 1200P varied between ±13 µm. Therefore, strength and modulus can be increased by treatment with velvet cloth and abrasive paper 1200P. On the contrary, the surface roughness of 0.1 wt % MLG/clay-EP nanocomposites treated with 320P varied between ±52 µm and that of abrasive paper 60P varied between ±103 µm. Therefore, it can be concluded that surface roughness beyond about ±20 µm has detrimental effect on tensile strength and modulus of 0.1 wt % MLG/clay-EP nanocomposites. Accordingly, the surface roughness below about ±20 µm is benign for tensile properties of 0.1 wt % MLG/clay-EP nanocomposites.

**Figure 19:**
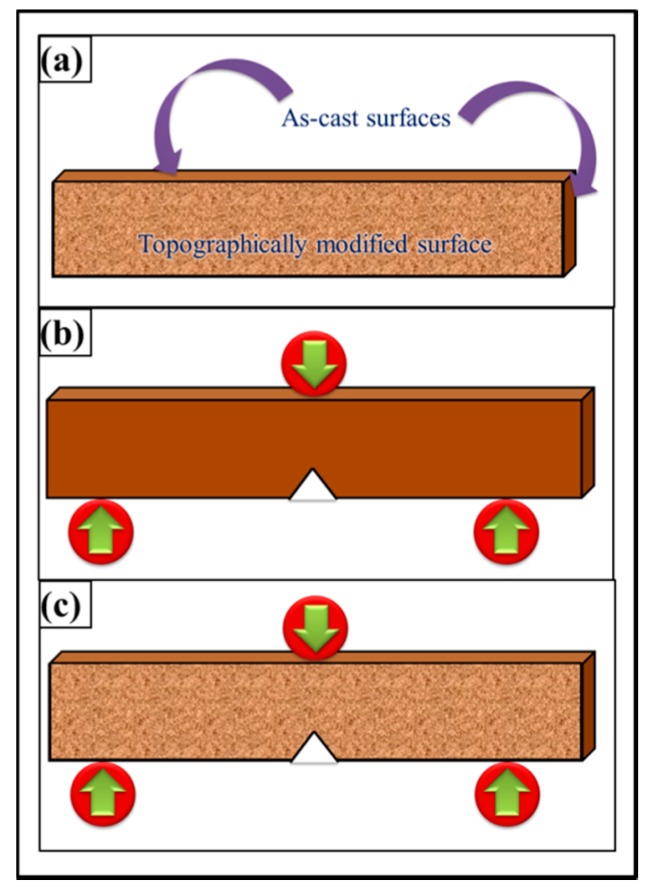
(**a**) Topographically modified front and back surfaces. The sides were not treated with abrasive papers; (**b**) As-cast; (**c**) topographically modified *K*_1C_ specimen under bending load.

The topographical features did not change the *K*_1C_ values in nanocomposites. One reason can be the orientation of topography with respect to notch and loading axis. The samples were treated with abrasive papers only along wider surfaces and not on the sides of the specimens. Therefore, when the samples were subjected to bending loading, the topographically treated surfaces were parallel to the axis of loading (Figure 19). The deformation and fracture takes place at the tip of notch whose size (3 mm) is much bigger than the surface roughness of topographically modified surfaces. These two reasons may contribute to topography (up to ±100 µm) having no influence on *K*_1C_.

**Figure 20:**
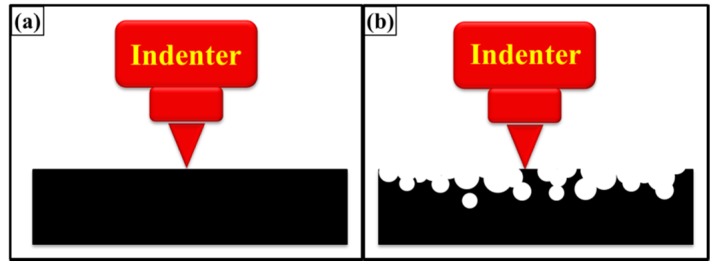
Indenter pressed against: (**a**) smooth; (**b**) rough surfaces.

This increase in hardness can be attributed to the smoothness of surfaces. A roughness was observed in as-cast specimens. When specimen contains corrugated surface, the tip of the indenter may or may not sit perfectly on the micro-plateau as shown in Figure 20. When the indenter sits perfectly on the surface, resistance is offered by the surface underneath and a high value of hardness is observed. On the contrary, when the indenter sits on the edge, lower resistance is offered by the edge and therefore, a lower value of hardness is observed. In samples treated with velvet cloth and abrasive paper 1200P, smoothness increased or at least sharp edges were removed. Therefore, an increase in hardness was observed. The hardness decreased in sample treated with abrasive papers 320P and 60P which shows that indenter faces less resistance due to corrugation of the surfaces. The hardness is also a function of applied load. It is known as indentation size effect according to which, “as load increases, the hardness decreases” [40]. It is also defined as, “as impression size decreases, the hardness increases”. Jiang *et al.* produced non-hydrogenated germanium carbide films by magnetron co-sputtering method in a discharge of Ar [41]. They observed that topography was significantly influenced by temperature and both hardness and Young’s modulus increased with increasing temperature. The compressive stress on the surface increases with increasing temperature [41]. Guo *et al.* deposited hydrogenated nanocrystalline silicon thin films with high hydrogen dilution ratio by plasma enhanced, chemical vapor deposition technique [42]. The surface roughness decreased with increasing the hydrogen dilution ratio. The hardness and Young’s modulus increased with finer topography [42]. A similar trend was observed in the current study where smooth surfaces (treated with velvet cloth and abrasive paper 1200P) caused an increase in modulus and hardness values. On the contrary, the coarser surfaces (treated with abrasive papers 320P and 60P) resulted in a decrease in modulus and hardness values. In metals, alloys and ceramics, the indentation size effect may be attributed to grain size, orientation and phases in the sample. In polymers, it may be because of the degree of crosslinking, orientation of polymer chains and size and distribution of porosity.

**Figure 21:**
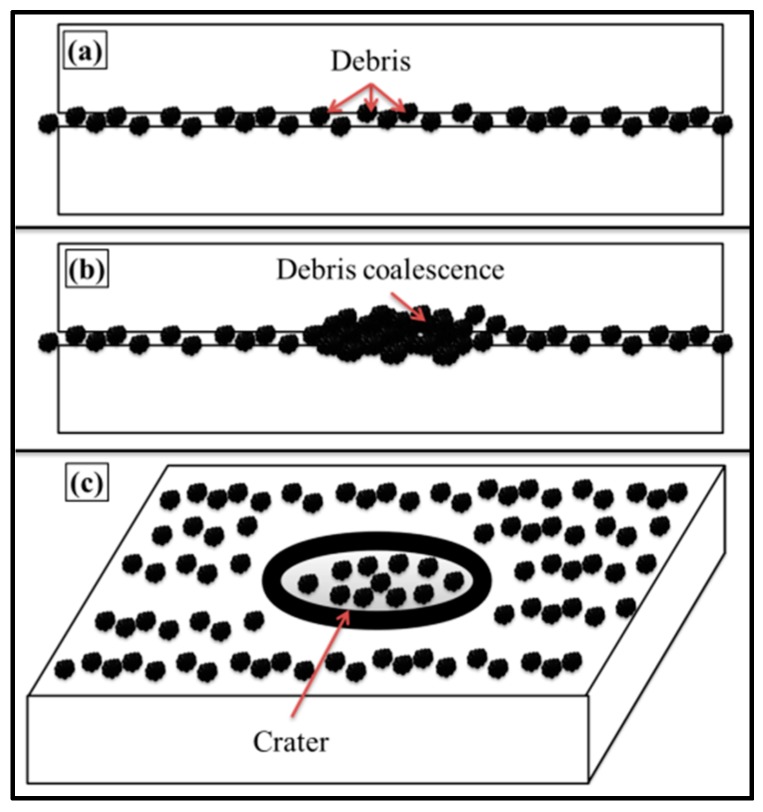
Debris are formed (**a**) which coalesce (**b**) and cause crater formation (**c**) due to frictional forces between two sliding surfaces.

It can be observed from the topographical analysis above that the treatment of 0.1 wt % MLG/clay-EP nanocomposites with abrasive papers produced topography with different size, shape and orientation of notches and corrugations. It can be attributed to varying roughness and particle size distribution of the abrasive papers used. Another important feasible mechanism could be the crater formation. When an abrasive tool slides against the sample surface, elevated temperatures are produced at the contact surface due to the frictional forces. The debris produced as a result of wear may coalesce at high temperature. This coalescence may result from diffusion, dissolution, cold-welding and related possible chemical interactions [43]. The coalesced particles result in a crater formation on the surface which may significantly deteriorate the mechanical properties as shown in Figure 21. If crater formation is part of the wear limit criterion, the topography can be analyzed by the depth of the crater or the projected area of the crater [43].

Surfaces are not completely flat at the microscopic level. At high magnification, even the best polished surface will show ridges and valleys, asperities and depressions. When two surfaces are brought together, they touch intimately only at the tips of a few asperities. At these points, the contact pressure may be close to the hardness of the softer material; plastic deformation takes place on a very local scale and cold welding may form strongly bonded junctions between the two materials. When sliding begins, these junctions have to be broken by the friction force and this provides the adhesive component of the friction. Some asperities may plow across the surface of the mating material and the resulting plastic deformation or elastic hysteresis contribute to the friction force; additional contributions may be due to wear by debris particles that become trapped between the sliding surfaces [44].

The smooth surfaces are necessary in sliding contact. When the surfaces contain high roughness and are slid against each other, wear takes place. The pointed debris formed as a result of wear, and if unable to find a place to escape, may cause cratering on the surface. The crater may act as a high stress concentration site due to the notch effect. This stress concentration may significantly deteriorate the mechanical properties of the bulk material. This effect may be further pronounced in the case of brittle polymers such as epoxy, as there are no intrinsic mechanism available to stop the crack advancement [10]. During sliding contact, thermal stresses are produced which can give rise to peculiar topographical features. In case of polymers, the thermal stresses may influence the degree of cross-linking. This altered degree of crosslinking at the surface may influence the overall mechanical properties of polymers [10]. In general, the mechanical properties improve with increased crosslinking. However, it was shown that fracture toughness decreases with increasing crosslinking [45].

The improvement in mechanical properties with the incorporation of MLG and nanoclay depend on many factors such as dispersion state and interfacial interactions. The maximum improvement in tensile strength is as high as 108% [46] and tensile modulus up to 103% [47]. Graphene was also found to improve flexural properties of nanocomposites. Naebe *et al.* produced covalent functionalized epoxy/graphene nanocomposites and reported an 18% and 23% increase in flexural strength and modulus, respectively [48]. Qi *et al.* produced graphene oxide/epoxy nanocomposites and reported an increase up to 53% in flexural strength [49]. The impact strength and hardness were also significantly improved by graphene in epoxy nanocomposites. For example, Ren *et al.* applied a combination of bath sonication, mechanical mixing and shear mixing to disperse GO in cyanate ester-epoxy and produced nanocomposites using in-situ polymerization [50]. They reported an increase of 31% in impact strength. Qi *et al.* produced graphene oxide/epoxy nanocomposites and reported an increase in impact strength up to 96% [51] whereas Lu *et al.* produced GO/epoxy nanocomposites and reported an increase in impact strength up to 100% [52]. Shen *et al.* produced GNS/epoxy nanocomposites and reported an increase in impact strength up to 11% [53] and Bao *et al.* reported an increase in hardness up to 35% [54]. The *G*_1C_ also improved with the incorporation of graphene in epoxy nanocomposites. Meng *et al.* produced epoxy/graphene nanocomposites and reported an increase in *G*_1C_ up to 597% [55].

The nanocomposites can be modeled and simulated to estimate the influence of nanofillers on mechanical, thermal and electrical properties of nanocomposites. Various theoretical and computational approaches have been employed to explore the effect of nanofillers on the performance of polymer nanocomposites, including but not limited to quantum mechanical-based methods [56], Continuum Mechanics (CM) [57], Molecular Mechanics (MM) [58], Molecular Dynamics (MD) [59], atomistic modeling [60], Density Functional Theory (DFT) [61] and multiscale modeling [62]. For example, some of the authors have shown that MLG is very efficient in scattering and dissipation of thermal flux in epoxy nanocomposites [26].

## 4. Conclusions

The 0.1 wt % MLG/clay-epoxy nanocomposites were treated with abrasive papers and the influence of topographical features was studied on mechanical and dynamic mechanical properties. It was observed that as-cast samples had a surface roughness which was reduced by treatment with velvet cloth and abrasive paper 1200P and increased by abrasive papers 320P and 60P. The maximum improvement in mechanical properties was observed when samples were treated with abrasive paper 1200P. The mechanical properties are a function of topographical features and also on the orientation of roughness with respect to loading axis. It can also be concluded that the surface roughness beyond ±20 µm has a detrimental effect on tensile strength and modulus of produced nanocomposites. Accordingly, the surface roughness below ±20 µm is benign for tensile properties. It was further observed that tensile properties are more sensitive to topography than flexural properties. The surface roughness beyond ±50 µm has a detrimental effect on flexural strength and modulus of produced nanocomposites. The tensile strength of as-cast 0.1 wt % MLG-EP nanocomposites is 60 MPa, while that of 0.1 wt % clay-EP nanocomposites is 49 MPa. A similar trend was observed for flexural properties and microhardness. Therefore, it can be concluded that MLG is more effective than nanoclay in improving the mechanical properties of epoxy. The tensile strength of as-cast 0.05 wt % MLG-0.05 wt % clay-EP nanocomposites is 61 MPa which is close to 1 wt % MLG-EP nanocomposites. Therefore, it can be concluded that synergistic effects are not that effective at low concentration of 0.1 wt % to cause a significant improvement in mechanical properties of produced nanocomposites. The topographical features did not change the dynamic mechanical properties. Therefore, it can be concluded that dynamic mechanical properties are impervious to topographical features of nanocomposites (at least up to ±100 µm).
